# Efficacy of 1 % silver sulphadiazine dressings in preventing infection of external fixation pin-tracks: a randomized study

**DOI:** 10.1007/s11751-015-0226-2

**Published:** 2015-08-15

**Authors:** Alfred O. Ogbemudia, Anirejuoritse Bafor, Ehimwenma J. Ogbemudia, Edwin Edomwonyi

**Affiliations:** Department of Orthopaedics and Trauma, University of Benin Teaching Hospital, PMB 1111, Benin City, Edo State Nigeria; Department of Medicine, University of Benin, Benin City, Edo State Nigeria; Department of Orthopaedics and Trauma, Irrua Specialist Teaching Hospital, Irrua, Edo State Nigeria

**Keywords:** 1 % silver sulphadiazine, Pin-skin interface, External fixation, Infection

## Abstract

Pin-track infection (PTI) is a common complication of external fixation. Antimicrobial dressings of the pin-site interface should reduce the severity and incidence of PTI. This study is aimed at determining the efficacy of 1 % silver sulphadiazine dressings in preventing PTI in external fixation. We compared the incidence of PTI between group A (dry sterile gauze dressing) and group B (1 % silver sulphadiazine impregnated gauze dressing). PTI was diagnosed when there was: (1) redness around any pin-site, (2) tenderness near a pin-site and (3) serous or purulent discharge from the pin-skin interface. With infection, swab was obtained for microscopy, culture and sensitivity. Pin-track infections were diagnosed in 22.5 and 4.1 % of patients in groups A and B, respectively. This difference was statistically significant. The commonest organism isolated from swabs was *Staphyloccus aureus*. In patients with external fixation, 1 % silver sulphadiazine lowered PTI. This further underlines the need for antimicrobial dressings of pin-sites. We recommend the use of 1 % silver sulphadiazine impregnated ribbon gauze for pin-site dressings.

*Level of evidence* II.

## Introduction

External fixation is integral to trauma and orthopaedic surgery. In addition to its use in stabilizing open fractures, the device is used in limb lengthening, bone transport and stabilization of the limb after corrective osteotomy and arthrodesis.

The pins used in external fixation maintain an open wound for bacterial invasion of tissues, making infection along the pin-track the most frequent complication of external fixation. The incidence of infection ranges between 11 and 96.6 % [1–6]. The first point of infection is the interface between the skin and pin. The infection then extends along the pin-track and, if allowed to continue, may lead to osteomyelitis, cellulitis and loosening of the pins. Even an infection as rare as myiasis has been reported [7].

Prevention of pin-track infection is, therefore, wise. Several studies describe varying methods for the control or prevention of infection. These methods include: technique of insertion such as prevention of thermal injury [8]; use of antimicrobial coated pins [9–11]; silver-coated pins [12]; and the use of antimicrobial impregnated patches [13].

In a related study, a combination of chlorhexidine and silver sulphadiazine in dressings was found very effective in reducing the incidence of pin-track infection [14].

Silver sulphadiazine is a very highly effective topical antimicrobial agent used in the dressing of burn wounds with the capacity to reduce bacterial colonization [15–17]. Silver sulphadiazine cream causes a slow and sustained release of silver ions which bind to bacterial deoxyribonucleic acid, inhibiting growth and multiplication of bacterial cells. It penetrates into exudates and necrotic tissue and is effective against *Staphylococcus aureus*, *Escherichia coli*, *Pseudomonas aeruginosa*, and strains of Proteus, Klebsiella, and *Candida albicans* [17]. The use of silver sulphadiazine in the management of extensive burns has not been found to be associated with systemic complications [18].

This longstanding safety and efficacy of silver sulphadiazine in the treatment of burns led to its choice as the agent for dressing of external fixator pins. A sterile ribbon gauze enmeshed in the creamy preparation of silver sulphadiazine and rolled around the skin-pin interface prolongs the antimicrobial activity of 1 % silver sulphadiazine at the pin-site and prevents bacterial colonization and subsequent infection. The potential here is to prevent infection to an extent similar to that from use of long-acting antimicrobial coated pins which are not available readily in the average clinical setting in most developing countries and are costlier than noncoated pins.

This study was designed to assess the efficacy of silver sulphadiazine dressings in the prevention of PTI by comparing the incidence of infection in a group dressed with 1 % silver sulphadiazine to another dressed with dry sterile gauze.

## Patients and methods

This study was conducted from January 2003 to December 2012 in a teaching hospital and two other hospitals in the same city. Institutional approval was obtained from the teaching hospital’s ethics and research committee.

All patients who gave consent (or had their guardians’ consent in the case of minors) to participate in the study were allocated to group A or B with the aid of a continuously updated register. Each nonconsenting patient received care with established treatment protocols. We excluded patients with chronic osteomyelitis or limb ischaemia lasting more than 8 h or established wound sepsis.

Ninety-eight patients were recruited consecutively into either group A or B. We recorded their clinical and demographic data. In determining the sample size, we planned to detect a double-fold reduction in the rate of pin-track infection amongst patients treated using silver sulphadiazine with a 90 % power of achieving 5 % significance. A sample size of 43 was determined per group. We made adjustment for a 12 % loss to follow-up. As a result, the final sample per group came to 49 patients. The resulting data were evaluated for statistical significance of the rate of infection using Chi-square test.

### Technique of application of pins

Each pin (a 4.5-mm Schanz screw) was applied through a 5-mm stab wound in the skin with a size 10 blade. The holes in the near cortex were pre-drilled with a hand drill using a 2.7-mm drill bit through a drill guide and the pins inserted until the far cortex was engaged. There was no pre-drilling for the insertion of 1.8 mm Kirschner wires for the Ilizarov circular external fixator. Each AO unilateral external fixators had four 4.5 mm Schanz screw pin-sites per limb; each unilateral rail had six 5 mm pin-sites, and each Ilizarov device had sixteen 1.8 mm pin-sites.

### Dressing protocol

Group A patients (the control group) had daily pin-site dressings with dry sterile strip of gauze after cleaning the pin-site with methylated spirit, while group B patients (the study group) had once-weekly pin-site dressing with a sterile strip of gauze that was impregnated with 1 % silver sulphadiazine cream (Dermazin^®^, Lek Pharmaceutical and Chemical Company, Ljubljana, Slovenia) and wound round the pin or wire after cleaning with methylated spirit. The immediate post-surgical dressings were removed 72 h, and fresh dressings were applied accordingly.

### Antibiotic prophylaxis

All the patients had intravenous ceftriazone 1 g daily for 48 h and metronidazole 500 mg 8-hourly for 24 h. Children were given 20 mg per kg of a single daily dose of ceftriazone and 7.5 mg/kg per dose of metronidazole. Our choice of post-operative antibiotics is based on the need for prophylaxis [19].

### Duration of hospital stay

All the cases were managed as inpatients for a minimum of 5 weeks and followed up for at least 16 weeks after removal of pins.

### Assessment of pin-sites

The presence or absence of pin-track infection was determined during dressing change at the ward round. Redness or tenderness near a pin (stage 1) or serous discharge (stage 2) or seropurulent or purulent discharge (stage 3) was taken as evidence of infection. The above classification set aside grades 4 and 5 of the Dahl classification [20] (4 = osteolysis requiring pin removal and 5 = ring sequestrum requiring debridement) which we presume are representative of a complicated infection. Swabs were taken from any pin-site with serous, seropurulent and purulent discharge for microscopy, culture and sensitivity. Antibiotic therapy was recommenced in those patients with pin-track infection.

## Results

There were 49 patients (49 fixators across forty-nine bones) in group A and 49 patients (51 fixators across fifty-one bones) in group B. The entire study group was made up of sixty-four males and thirty-four females with an average age of 37.2 ± 15.8 years (range 4–75 years). There were 33 males and 16 females in group A with an average age of 36.7 ± 16.8 years (a range of 7–75 years) as well as 31 males and 18 females in group B with an average age 37.7 ± 15.2 years (a range of 4–75 years). There was no loss to follow-up within 16 weeks after removal of pins.

Tables 1 and 2 show the details of demographic and clinical data of the study groups. The external fixators remained in place for an average of 9.8 weeks in group A and 13.2 weeks in group B. Eleven patients (22.5 %) in group A and two (4.1 %) in group B had pin-track infections, respectively (*p* = 0.01). Thirty-eight pin-tracks (14.2 %) were infected in group A, while seven pin-tracks (1.9 %) were infected in group B (*p* < 0.01). The types of fixation, number of pin-sites and infection rates are shown in Table 3.

**Table 1:** The demographic characteristics of the study groups

Feature	Control group (A)	Study group (B)
No of patients	49	49
Age in years (range)^a^	36.7 ± 16.8 (7–75)	37.7 ± 15.2 (4–75)
Male/female	33:16	31:18

^a^Mean ± standard deviation and (range)

**Table 2:** Site of fixation and indication for external fixation

Feature	Control group (A)	Study group (B)
*Site of external fixation*		
Humerus	7	6
Radius	2	2
Ulna	2	1
Hand	1	0
Femur	5	11
Tibia	29	31
Foot	2	0
Ankle	1	0
Total	49	51
*Indication* (*diagnosis*)		
Gunshot Injury	14	17
Road traffic accident	32	28
Achondroplasia	1	1
Blount disease	2	3

**Table 3:** Types of fixation, number of pin-sites and infection rates

Feature	Control group (A)	Study group (B)
Number of pin-sites	268	368
AO tubular external fixation	43	41
Unilateral rail	0	2
Ilizarov device	6	8
Pin tract infection (patients)^a^	11 (22.5 %)	2 (4.1 %)
Pin tract infection (pin-sites)^a^	38 (14.2 %)	7 (1.9 %)

^a^Number and (percentage)

All infections were superficial in both groups. There was no difference in the two groups in terms of pin complications post-removal.

## Discussion

The incidence of infection amongst patients who had dry dressings (22.5 %) against those who had silver sulphadiazine dressings (4.1 %) indicates that the antimicrobial effect of silver dressing is efficacious. This significantly lower incidence of infection inpatients who had silver sulphadiazine dressings is attributable to the antimicrobial qualities of 1 % silver sulphadiazine in controlling microbial invasion of the pin-site since both control and study groups were given the same dose of antibiotics for the same duration and had cleaning with methylated spirit before application of dressings. The inpatient care offered to the patients eliminated the confounding influence of environmental and related caregivers’ factors on the results.

Infection at the pin-site from external fixation may lead to loosening, early discontinuation of external fixation and systemic sepsis in some cases. Loosening and early discontinuation of external fixation would mar any procedure despite being well done.

The results of this study support several others which have found antimicrobial preparations effective in preventing pin-track infection [9–12, 14]. The value of antimicrobial preparations has been demonstrated by use of chlorhexidine coating in controlling microbial colonization of epidural and central venous catheters [21–23]. Similarly, the reduction in incidence of PTI in this study is attributed to the antimicrobial activity provided by the 1 % silver sulphadiazine and is consistent with the efficacy of silver-coated pins in reducing pin-track infection.

The choice of once-weekly dressings was determined by a need to have a dressing routine that would be feasible in a busy unit that has to maintain time for other aspects of patient care. This choice is supported by an earlier study showing no difference in outcome between daily and weekly dressing changes in the incidence of pin-track infection [24]. Use of an effective antimicrobial dressing has been described previously to be as effective as systemic antibiotic administration in preventing pin-track infections [25].

Whilst self-applied nonantimicrobial based dressings are feasible, it is associated with a high incidence of pin-track infection [26]. The possibility of adding 1 % silver sulphadiazine cream once or twice daily to the pin-site interface (PSI) at home by a patient has potential to be an effective solution in cases where home dressings are associated with increased incidence of infection. The outcome of this study parallels the established efficacy of silver sulphadiazine in burn wound management [17, 18].

There are limitations to this study. We were unable to blind patients and caregivers to the type of dressing used, and the prolonged admission in hospital in order to eliminate environmental factors may influence pin-site contamination. This absence of blinding did not seem to have had a significant influence on the results because the diagnosis of infection was determined during dressing changes on ward rounds using the outlined criteria.

## Conclusion

The incidence of pin-track infection was significantly reduced by the use of 1 % silver sulphadiazine cream impregnated gauze dressings at the pin-site. There was no significant difference in complications after pin or wire removal. On the basis of our findings, we recommend the use of 1 % silver sulphadiazine dressings for external fixator pins.
